# 

**Published:** 1891-01

**Authors:** 


### CONTENTS

### ORIGINAL COMMUNICATIONS—

President's Address before the Southern Surgical and Gynecological Society, Atlanta, November 11, 1890 ........ 643

Inguinal Colotomy, with Report of a Case. By Arch. Dixon, M. D., Henderson, Ky ........ 660

A Case of Placenta Prævia, with Faulty Position of the Child. By T. E. Nott, Jr. ........ 672

Creosote in Treatment of Phthisis. By C. E. Murphey, M. D., Atlanta, Ga ........ 674

Nasal and Pharyngeal Manifestation of Syphilis—Result and Treatment. By J. G. Carpenter, M. D., Stanford, Ky ........ 677

One Hundred Consecutive Cases of Skin Disease. By M. B. Hutchins, M. D., Atlanta, Ga ........ 684

### CORRESPONDENCE—

Our New York Letter ........ 688

### EDITORIAL—

Announcement ........ 694

Preachers, Patent Medicines and Doctors' Bills ........ 695

Bogus Diplomas ........ 696

Koch's Lymph ........ 697

Atlanta Society of Medicine ........ 697

The Clinic ........ 697

The Mattison Prizes ........ 698

Hydronaphthol as an Antiseptic ........ 698

Fracture of Lower End of Radius ........ 699

Diphtheria ........ 699

The University Medical Magazine ........ 699

Pharmacology and Therapeutics ........ 700

Notes upon Somnal, the New Hypnotic ........ 701

The Rationale of Influenza ........ 705

Index to Advertisements ........ XII

Reading Notices ........ X-XI

Premium List ........ XXXVI-XXXVII

